# The cationic amino acid transporter 2 is induced in inflammatory lung models and regulates lung fibrosis

**DOI:** 10.1186/1465-9921-11-87

**Published:** 2010-06-24

**Authors:** Kathryn A Niese, Monica G Chiaramonte, Lesley G Ellies, Marc E Rothenberg, Nives Zimmermann

**Affiliations:** 1 Division of Allergy and Immunology, Cincinnati Children's Hospital Medical Center, 3333 Burnet Ave, Cincinnati, Ohio 45229, USA; 2 Division of Immunobiology, Cincinnati Children's Hospital Medical Center, 3333 Burnet Ave, Cincinnati, Ohio 45229, USA; 3 Cancer Center and Department of Medicine, University of California San Diego, 3855 Health Sciences Drive, La Jolla, California 92093, USA; 4 Department of Pediatrics, University of Cincinnati College of Medicine, 231 Albert Sabin Way, Cincinnati, Ohio 45267, USA

## Abstract

**Background:**

Arginine is an amino acid that serves as a substrate for the enzymes nitric oxide synthase (NOS) and arginase, leading to synthesis of NO and ornithine, respectively. As such, arginine has the potential to influence diverse fundamental processes in the lung.

**Methods:**

We used mice deficient in cationic amino acid transporter (CAT) 2 in models of allergic airway inflammation and pulmonary fibrosis.

**Results:**

We report that the arginine transport protein CAT2 was over-expressed in the lung during the induction of allergic airway inflammation. Furthermore, CAT2 mRNA was strongly induced by transgenically over-expressed IL-4, and allergen-induced expression was dependent upon signal-transducer-and-activator-of-transcription (STAT) 6. *In situ *mRNA hybridization demonstrated marked staining of CAT2, predominantly in scattered mononuclear cells. Analysis of allergic airway inflammation and bleomycin-induced inflammation in CAT2-deficient mice revealed that while inflammation was independent of CAT2 expression, bleomycin-induced fibrosis was dependent upon CAT2. Mechanistic analysis revealed that arginase activity in macrophages was partly dependent on CAT2.

**Conclusion:**

Taken together, these results identify CAT2 as a regulator of fibrotic responses in the lung.

## Background

Recent studies have implicated amino acids, specifically tryptophan and arginine, in the regulation of immunity and tolerance. Elegant studies demonstrated an important role for tryptophan metabolism through indoleamine 2,3-dioxygenase (IDO) in inhibition of experimental asthma [1]. However, the role of arginine transport and metabolism remains unclear. Intracellular arginine is metabolized by both the nitric oxide synthase (NOS) and arginase pathways. The product of the former, NO, has been implicated in the regulation of both inflammation and airway tone. Similarly, products of the arginase pathway, such as ornithine, are regulators of key processes involved in lung inflammation, including cell hyperplasia and collagen deposition [2,3]. Among the transport systems that mediate L-arginine uptake, cationic amino acid transporters (CAT1, -2 or -3) are considered to be the major arginine transporters in most cells and tissues [4]. We chose to focus on CAT2 because of its essential role in arginine transport in immune cells, including macrophages [5]. Defining the role of arginine and its transport protein CAT2 has been aided by the generation of CAT2-deficient mice [5]. While these mice are grossly normal, their peritoneal macrophages have a 95% decrease in L-arginine uptake and a marked impairment in NO production [5,6]. In contrast, CAT2-deficient fibroblasts have largely intact NO production [7]. Our studies demonstrated that CAT2 is an essential part of the host protective immune apparatus in the lung in that CAT2-deficient mice displayed baseline inflammation [8], identifying CAT2 as responsible for maintenance of inflammatory homeostasis. A recent publication demonstrated that CAT2-deficient mice are significantly more susceptible to the parasite *T gondii *and develop enhanced fibrosis and granuloma formation in response to *S mansoni *[9]. Since arginine entry into the NOS and arginase pathways could have multiple effects, both positive and negative, on lung processes during pathological conditions (e.g. inflammation and fibrosis), we used CAT2-deficient mice to test the net effect of reducing transport of arginine in experimental asthma and experimental lung fibrosis.

## Methods

### Mice

All animal studies were approved by the Cincinnati Children's Hospital IACUC committee. Mice were bred "in house" in specific pathogen-free conditions. CAT2-deficient [5], STAT6-deficient [10] and IL-4 transgenic [11] mice were described previously. CAT2-deficient mice were either on the FVB/N or C57Bl/6 background. Both strains have been backcrossed for more than 10 generations.

### Induction of experimental disease models

Mice were allergen challenged as described previously [12-14]. Briefly, mice were sensitized intraperitoneally (i.p.) with ovalbumin (OVA, 100 μg) in alum (1 mg) and challenged intranasally (i.n.) with 50 μg OVA or saline. After instillation, mice were held upright until alert. Mice were sacrificed 18-24 hours following the last challenge. For the bleomycin model, mice were treated with a single dose (0.03 U/mouse) of Bleomycin intratracheally (i.t.) and sacrificed 14 days later. Bronchoalveolar lavage was performed, and cells were counted by hemocytometer and differentiated based on morphology following Diff-Quick staining of cytospin preparations.

### *In situ *hybridization of mouse lung

*In situ *hybridization was performed as described [13]. In brief, murine CAT2 cDNA was subcloned from Image Consortium clone 5344352 into pBluescript, linearized by Hind III and Not I digestion, and anti-sense and sense RNA probes, respectively, were generated by T3 and T7 RNA polymerase (Riboprobe Gemini Core System II transcription kit; Promega, Madison, WI). The radiolabeled [αS^35^-UTP] probes were hybridized and washed under high-stringency conditions.

### Northern blot analysis

RNA was extracted using the Trizol reagent as per the manufacturer's instructions. The cDNA probe, generated from commercially available vectors [Image Consortium clone 5344352 in pCMV-SPORT6 obtained from American Tissue Culture Collection, Rockville, MD], was liberated with NdeI and MluI, confirmed by sequencing, radiolabelled with ^32^P, and hybridized using standard conditions, as described previously [13].

### Measurement of collagen accumulation

Collagen accumulation was determined by measuring the content of hydroxyproline as previously described [8]. Additionally, fibrosis was evaluated histologically. Tissues were fixed in 10% neutral buffered formalin and paraffin embedded. Sections were stained with Masson's trichrome to evaluate fibrosis. Scoring (0-3) represents evaluation of intensity and extent of fibrosis determined by an observer blinded to treatment and genotype.

### Arginase activity

Arginase activity in intact cells (peritoneal macrophages from naïve mice) was measured using incorporation of radioactive arginine as previously described [15]. Cells (2 × 10^5 ^cells/100 μl PBS and 22 mM glucose/well of 96-well-plate) were incubated with ^14^C-arginine (L-[guanido-^14^C], NEN) for 18 hours, and ^14^C-urea present in the cell supernatant was metabolized with urease. Released ^14^CO_2 _was collected and measured by scintillation counting. Arginase activity in lysed macrophages was measured using the blood urea nitrogen reagent (Sigma Chemical Company, St. Louis, MO) according to established techniques [16-18].

### Cytokine levels

Cytokine levels were determined by Multiplex analysis on BALF supernatant samples. Analysis was performed by Millipore using the mouse 32 cytokine/chemokine panel.

### Statistical analysis

Values are reported as the mean ± standard deviation. The significance of differences between groups were analyzed by ANOVA using Prism software. Pairwise comparisons were performed by Student's t-test. Differences are considered significant if P < 0.05. Trichrome staining quantification scoring was tested by Mann-Whitney U test.

## Results

### CAT2 is an allergen-induced gene in experimental asthma

**Figure 1:**
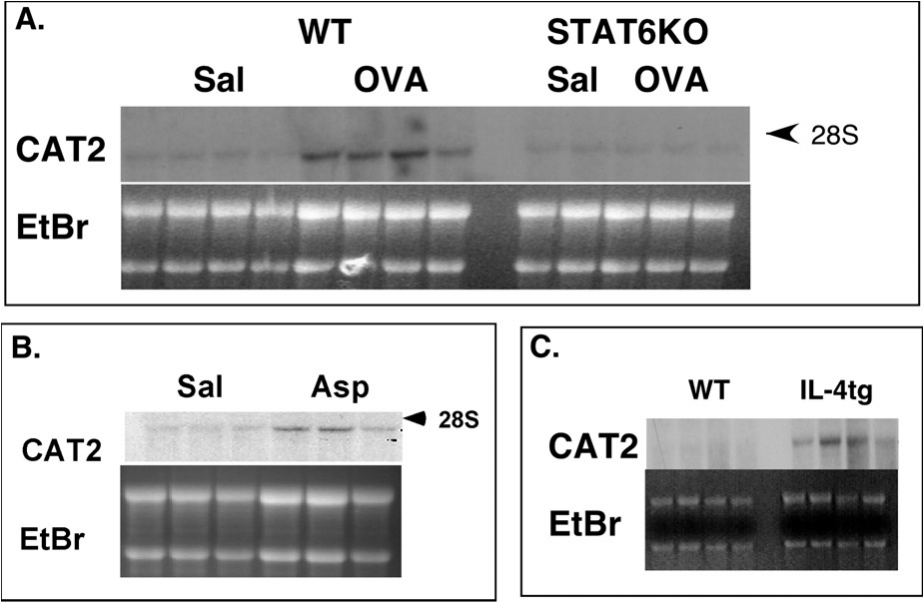
**CAT2 induction by allergen challenge**. In (A), wild type and STAT6-deficient mice were allergen challenged with OVA or saline (Sal) and CAT2 expression was determined by Northern blot analysis. In (B), the expression ofCAT2 following intranasal challenges with Asp or saline is shown. In (C), CAT2 expression in wild type and IL-4 lung transgenic mice was determined by Northern blot hybridization. The 4.5 kb band of CAT2 mRNA is shown. The Ethidium bromide (EtBr)-stained gel is also shown. Each lane represents whole lung RNA from an individual mouse.

Using microarray analysis, we identified a set of "asthma signature" genes that provided a valuable opportunity to define new pathways involved in the pathogenesis of allergic airway inflammation [13,14]. This analysis was performed on mice with experimental asthma induced by two distinct protocols. In one protocol, mice were sensitized using OVA and the adjuvant alum and subsequently challenged intranasally with OVA or saline as control. In other experiments, both sensitization and challenge were achieved with the antigen *Aspergillus fumigatus *intranasally. We were struck by the high level of transcripts for cationic amino acid transporter 2 (CAT2) in the asthmatic lung compared to saline-challenged lungs. We chose to focus on this gene since arginine transported by CAT2 can be metabolized by the arginase and NOS pathways, both of which may have a profound effect on inflammation. Interestingly, microarray analysis revealed very specific expression of CAT2 compared with other CAT genes. For example, the hybridization signals for CAT1 and CAT3 were not detectable in the saline- and allergen-challenged lung (data not shown). We substantiated these findings by Northern blot analysis. In contrast to the saline-challenged control mice which had low levels of CAT2, mice challenged with OVA or Aspergillus had significantly increased levels of CAT2 mRNA (Figure 1A and 1B, respectively). We were next interested in testing the hypothesis that overexpression of IL-4, particularly in the lungs, was sufficient for induction of CAT2. Mice that overexpress the IL-4 transgene in pulmonary epithelium (under the control of the Clara cell 10 promoter) have several features of asthma including eosinophil-rich inflammatory cell infiltrates, mucus production, and changes in baseline airway tone [11]. Indeed, IL-4 lung transgenic mice had a marked increase in the level of CAT2 (Figure 1C). IL-4 signaling often involves the transcription factor STAT6. Thus, we hypothesized that allergen-induced CAT2 expression may be dependent on STAT6. Wild type and STAT6-deficient mice were challenged with OVA, and CAT2 expression was detected by Northern blot analysis. As seen in Figure 1A, CAT2 expression was significantly decreased in STAT6-deficient allergen-challenged mice, compared to wild type allergen-challenged mice. In summary, we show that CAT2 expression is induced by allergen in a STAT6-dependent manner.

### CAT2 *in situ *hybridization

**Figure 2:**
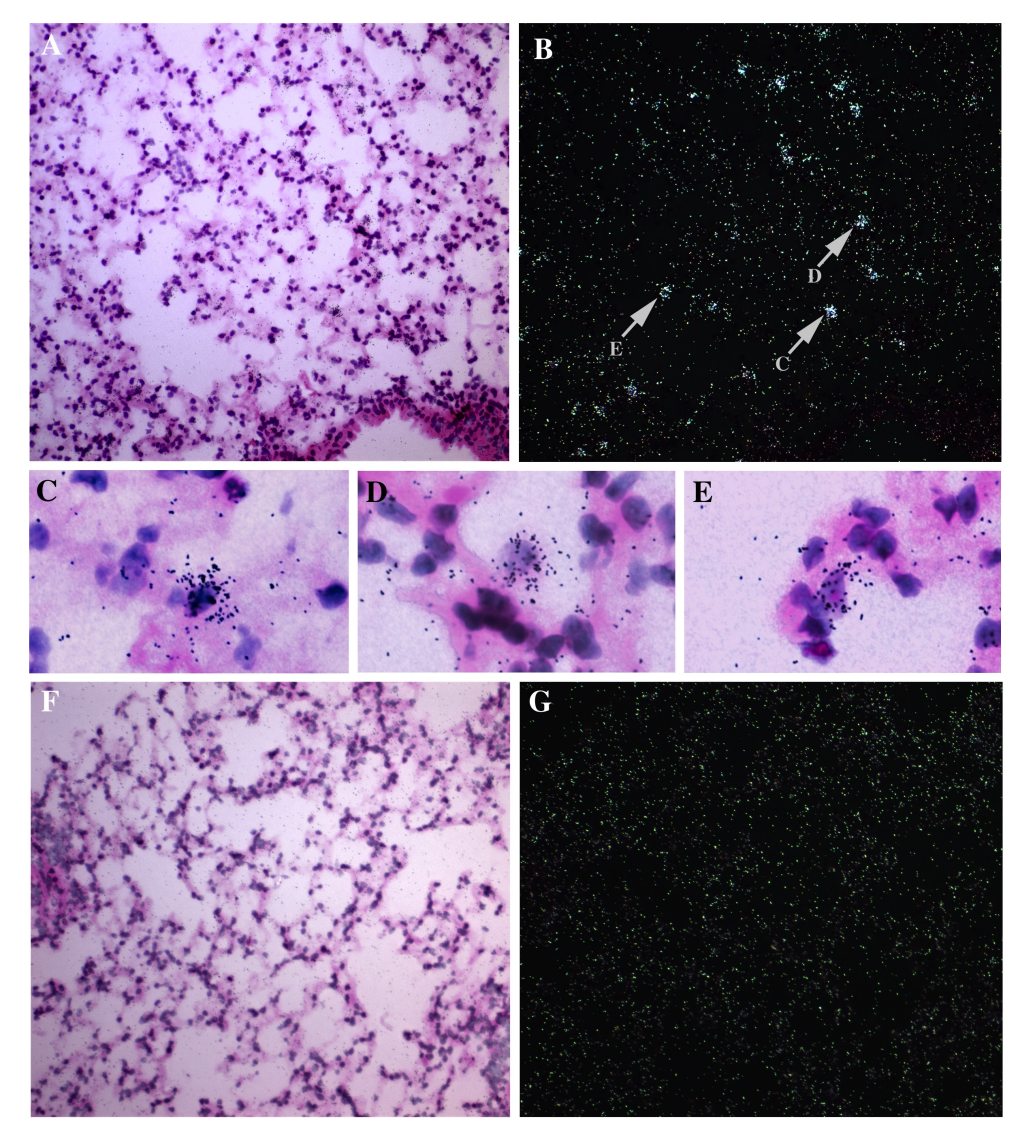
***In situ *hybridization**. Expression of CAT2 in OVA-challenged mice was evaluated by *in situ *hybridization. Bright-field (A) and dark-field (B) images from an OVA-challenged mouse are shown. Panels C-E show close-ups of individual positive cells to allow for evaluation of morphology. Panels F and G are OVA sense control.

To begin to address the distribution of CAT2 mRNA-positive cells in the allergic lung, we performed *in situ *hybridization for CAT2 mRNA. OVA/alum sensitized mice were challenged with intranasal OVA or saline, and *in situ *hybridization was performed on lung tissue obtained 18 hours after the second allergen or saline challenge. Anti-sense staining of OVA-challenged lungs revealed strong CAT2 mRNA expression in individual scattered mononuclear cells, most consistent with resident macrophages (Figure 2A-E). There was no staining of the epithelial cell lining. Identifiable eosinophils appeared negative. There was no signal in the smooth muscle cells of the bronchial airways or arterioles, alveolar lining cells or endothelial cells. As controls, anti-sense and sense staining of the saline-challenged lung revealed no detectable staining (data not shown). Additionally, no specific staining was observed when the OVA-challenged lungs were stained with the sense probe (Figure 2F-G).

### CAT2 expression is not required for allergen-induced lung inflammation

**Figure 3:**
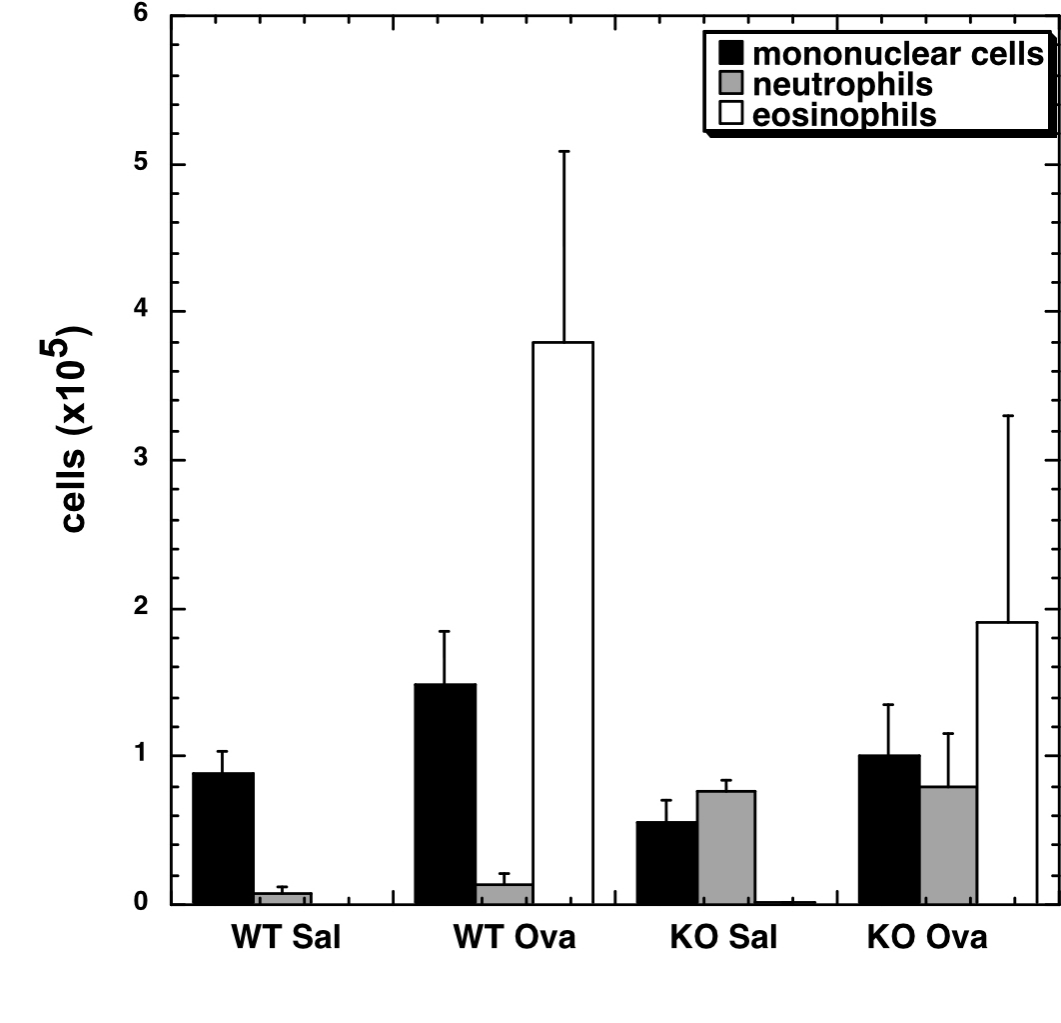
**Allergen challenge of CAT2-deficient mice**. Wild type and CAT2-deficient mice were challenged with OVA. Infiltration of inflammatory cells in the bronchoalveolar lavage fluid is shown. Representative of 3 experiments with 3-4 mice/group is shown.

L-arginine transported by CAT2 can be metabolized by the arginase and NOS pathways, both of which can have a profound effect on inflammation. In order to test the hypothesis that CAT2 is required for allergen-induced inflammation, we challenged wild type and CAT2-deficient mice with OVA and measured the final inflammatory endpoint: cell recruitment and composition in the bronchoalveolar lavage fluid (BALF). As seen in Figure 3, allergen-induced inflammation was comparable in wild type and CAT2-deficient mice. The level of systemic sensitization, as measured by OVA-specific IgG1, was comparable between wild type and CAT2-deficient mice (n = 4 experiments, data not shown). Saline-challenged CAT2-deficient mice had baseline inflammation (primarily neutrophilia) as we have previously reported [8]. In summary, our data demonstrate appropriate inflammatory responses in the lung in response to allergic stimuli.

### CAT2 expression is required for collagen deposition following bleomycin exposure

**Figure 4:**
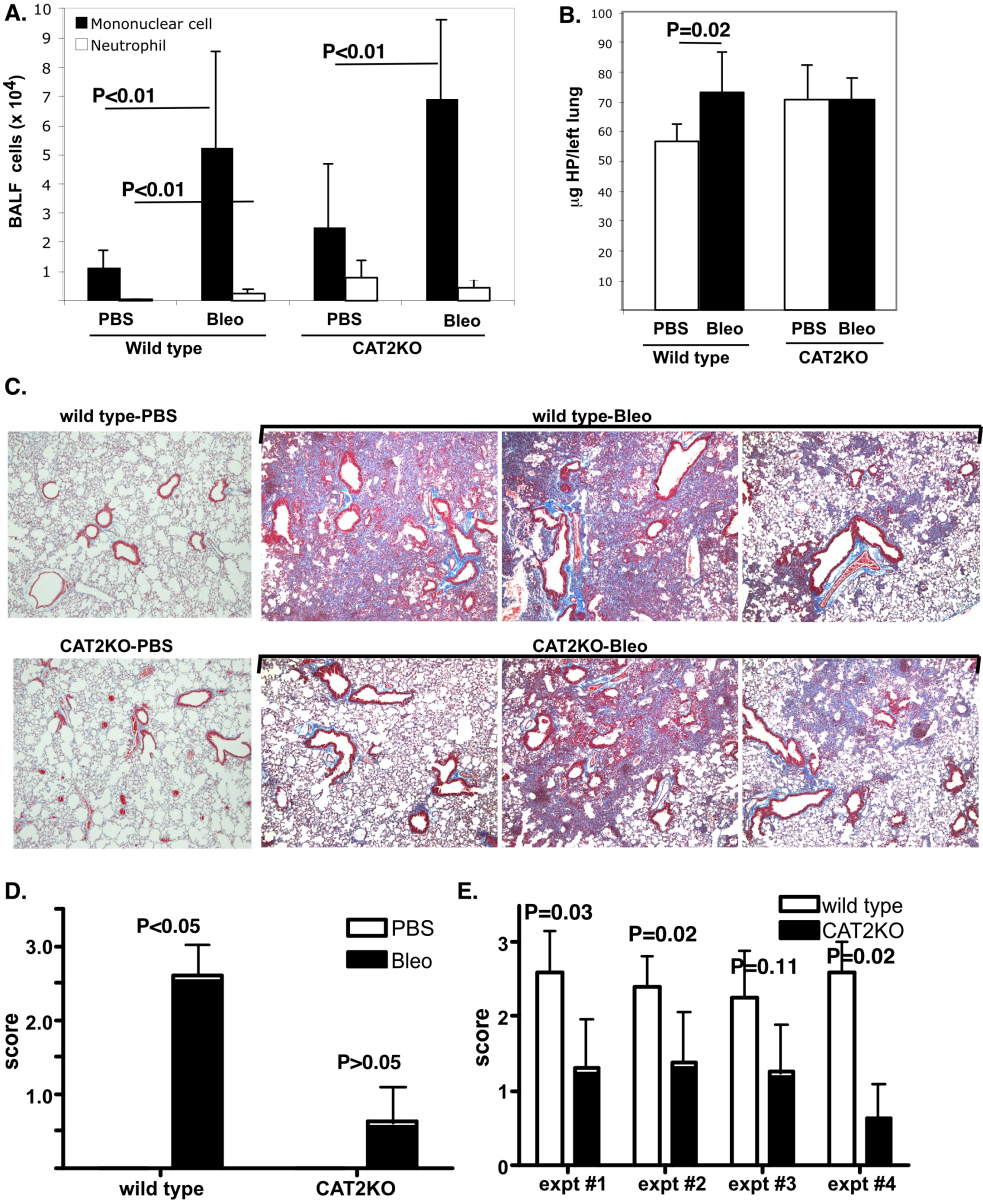
**Bleomycin treatment of CAT2-deficient mice**. Wild type and CAT2-deficient mice (C57Bl/6 background) were challenged with bleomycin (bleo) i.t. and BALF cells (A) and hydroxyproline levels (B) were measured 14 days later. Representative of 3 experiments is shown. Data are mean ± standard deviation of 5-6 mice/group. In (C), representative photomicrographs of 3 individual mice stained with trichrome staining are shown (WT-wild type; KO- CAT2-deficient mice). In D and E, quantification of trichrome staining in bleomycin-treated mice in a representative (D) and four individual experiments (E) is shown (one using mice on C57Bl/6 background and 3 on FVB/N background). Lungs were scored on a scale from 0-3 for intensity and extent of involvement. (D) P values by Kruskal-Wallis with Dunn's Multiple Comparison Test. (E) n = 4-8 mice/group (only bleomycin-treated groups are shown) in each experiment with a total of 19 wild type and 21 CAT2-deficient mice. P values shown are by Mann-Whitney test.  2-way ANOVA (variables: genotype and experiment) identified genotype as the statistically significant (P < 0.0001) source of variation (interaction and experiment P = not significant).

Since CAT2 was not involved in inflammation, we hypothesized that it would be involved in lung fibrosis. This is based on the following: 1) CAT2 is upregulated in bleomycin-induced pulmonary fibrosis [19] and 2) arginase metabolizes arginine to ornithine, which can be further metabolized by ornithine aminotransferase to proline, an amino acid that is often the rate-limiting substrate for collagen synthesis. In order to test this hypothesis, we used a strong, well-established model of pulmonary fibrosis and treated CAT2-deficient mice with bleomycin i.t.: this leads to chronic inflammatory infiltrate in the alveolar spaces, mostly composed of macrophages and lymphocytes, as seen in Figure 4A. Similar induction of inflammation is seen in CAT2-deficient mice treated with bleomycin suggesting that CAT2 is not required for the inflammatory process, similar to what was observed with allergen-induced lung inflammation (Figure 3). In support of these data, multiplex analysis of cytokines and chemokines in the BALF identified that multiple cytokines/chemokines (including IP-10, MIG, IL-6, LIF and G-CSF) are induced by bleomycin treatment at comparable levels in wild type and CAT2-deficient mice (data not shown).

In order to address the role of CAT2 in collagen deposition, we measured hydroxyproline levels in the lungs of bleomycin-treated mice. As seen in Figure 4B, CAT2-deficient mice had increased baseline levels of hydroxyproline (P = 0.02) as we have previously reported [8]. However, while wild type mice responded to bleomycin with increased levels of hydroxyproline, CAT2-deficient mice were unable to further increase lung fibrosis. In order to assess the fibrosis by an independent method, we stained lung tissue sections by trichrome staining. As seen in Figure 4C, both wild type and CAT2-deficient mice had areas of fibrosis and tissue consolidation accompanied by inflammation, which is typical of day 14 lung sections in bleomycin-induced fibrosis. When sections were scored for intensity and extent of involvement by an observer blinded to treatment, the level of lung tissue fibrosis/consolidation was significantly decreased in CAT2-deficient mice compared with wild type mice (Figure 4D and 4E). Control mice of either genotype did not show histological evidence of fibrosis/consolidation or inflammation (Figure 4C, D and data not shown).

### CAT2 requirement for macrophage arginase activity

**Figure 5:**
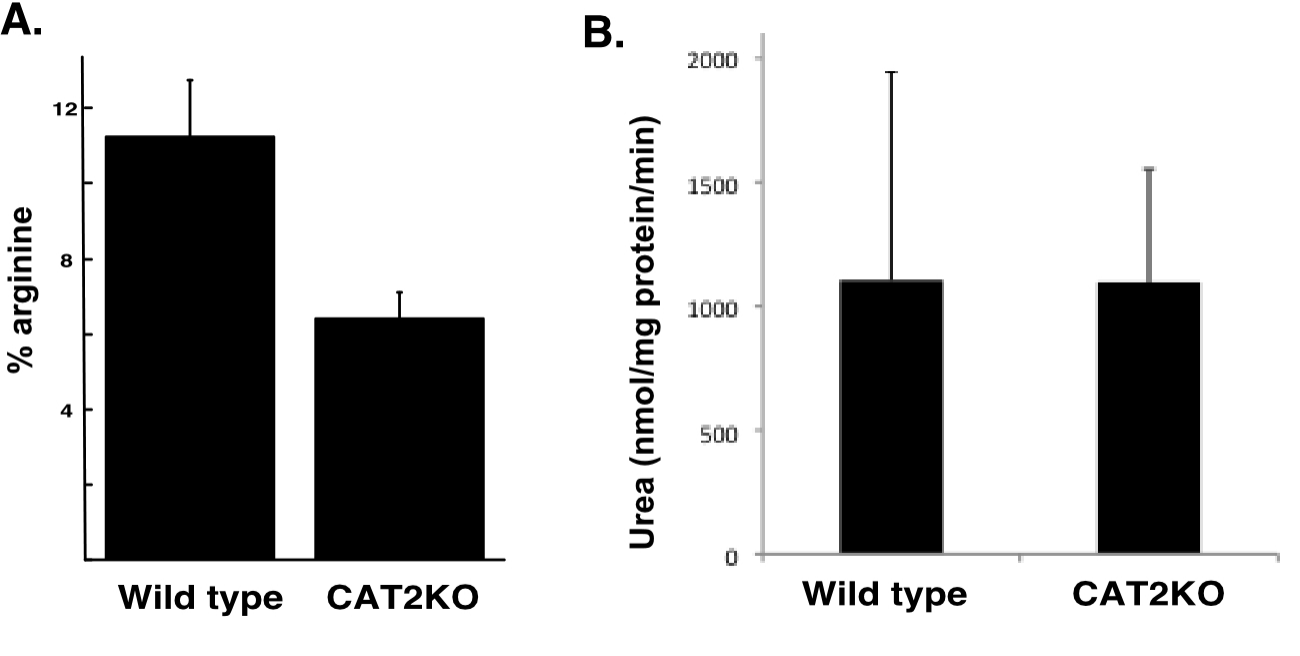
**Role for CAT2 in macrophage arginase activity**. In A, peritoneal macrophages were isolated from CAT2-deficient (CAT2KO) and wild type mice and arginase activity was measured by hydrolysis of extracellularly added ^14^C-arginine by intact macrophages. A representative experiment (out of 5 total; 3 with mice on C57Bl/6 and 2 on FVB/N background) is shown. Data are mean ± standard deviation of triplicate measurements. In (B), macrophages from wild type and CAT2-deficient mice had arginase activity measured following cell lysis by detergent.

CAT2 may regulate fibrotic processes via arginase activity. Indeed, arginase expression is increased in the lungs of bleomycin-treated mice [19]. Thus, we tested the hypothesis that CAT2 is required for arginase activity in intact macrophages by using the whole cell arginase activity assay [15]. Peritoneal macrophages from naïve wild type and CAT2-deficient mice were collected, and arginase activity was measured. There was a 52.3 ± 36.4% (P = 0.02, n = 3 experiments performed in triplicate) decrease in arginase activity in CAT2-deficient mice compared to strain-matched C57BL/6 control mice. A representative experiment is shown in Figure 5A. Similar results were obtained with FVB/N mice (60% decrease, n = 2 experiments performed in triplicate). Importantly, when arginase activity was measured using the lysed-cell method as a measure of arginase expression and activity in cells irrespective of arginine transport into cells, there was no difference between wild type and CAT2-deficient macrophages (arginase activity in CAT2-deficient macrophages was 93.7 ± 17.6%, P = 0.69, n = 3 experiments) (Figure 5B). These data indicate that arginase activity is partially dependent on arginine transport through CAT2 in peritoneal macrophages.

## Discussion

Recent studies have suggested a role for arginine transport and metabolism in a variety of inflammatory disorders. In this report, we used CAT2-deficient mice to test the role of arginine transport in lung inflammatory conditions, including experimental asthma and bleomycin-induced lung fibrosis. Our studies revealed several novel findings. First, we demonstrate that CAT2 mRNA expression is induced in experimental asthma and that the main cell type expressing it is a mononuclear cell most consistent with alveolar macrophages. Second, we demonstrate that CAT2 is required for bleomycin-induced pulmonary fibrosis. Third, in contrast to the fibrosis data, we show that CAT2 expression is not required for lung inflammation in either allergen-induced or bleomycin-induced models. Finally, we show that CAT2 is partially required for arginase activity in macrophages, which may contribute to the development of fibrosis.

Even though CAT2 is induced highly in asthma, it is not required for experimental asthma-induced inflammation. This observation is consistent with our recent finding that arginase is not required for allergic airway inflammation, despite high level of induction [20]. In contrast, CAT2 was recently shown to have an important, host-protective role in parasitic infestation models [9], similar to studies which demonstrated a role for arginase expression in host defense in models of parasite infestation (including Schistosome mansoni, Heligmosomoides polygyrus, Leishmania sp, Toxoplasma gondii and Nipostrongylus brasiliensis) [21-26]. In summary, our data suggest a divergent role for arginase I and CAT2 in allergic inflammation compared to parasitic responses. Since arginase and CAT2 are prominent products of alternatively activated macrophages, which are induced by IL-4 in both allergic and parasitic responses, our data suggest that alternatively activated macrophages evolved to combat parasitic infections and are either bystanders in allergic inflammation or have developed other effector molecules for allergic Th2-associated responses. In contrast, we have previously demonstrated that CAT2 has a suppressive role in baseline inflammation [8]. We speculate that resident immunosuppressive macrophages require CAT2 for the maintenance of inflammatory homeostasis in the lung, whereas highly activated M2 macrophages express CAT2 but do not require it for the inflammatory process.

Similarly, the role of CAT2 in fibrosis is context-dependent. Baseline fibrosis is increased in CAT2-deficient mice as measured by the whole lung hydroxyproline assay [8]. Interestingly, this baseline fibrosis is not evident histologically by trichrome staining, especially when slides are scored by an observer blinded to genotype and treatment. We did not assess allergen-induced fibrosis because fibrosis does not develop in the acute OVA-induced model. However, we used a well-characterized model of bleomycin-induced fibrosis, which has previously been shown to exhibit induced transcription of CAT2 [19]. Interestingly, in this model fibrosis is dependent on CAT2 as measured by either whole lung hydroxyproline measurement or blinded quantification of trichrome staining. Since previous studies have shown that proline synthesis (often a rate-limiting step in collagen synthesis) is downstream of CAT2 and arginase, we tested the hypothesis that CAT2 is required for arginase activity in macrophages. We found a notable requirement, suggesting a possible role for arginase in the CAT2-mediated fibrosis.

The requirement for CAT2 for arginase activity in macrophages is controversial. Thompson et al. [9] demonstrated that CAT2-deficient macrophages and fibroblasts have increased arginase activity. However, their assay was performed with ruptured cells and exogenously added arginine (250 mM; Km for arginase is ~5 mM) thus circumventing the function of arginine transport into the cell. Yeramian et al. [27] demonstrated decreased arginine catabolism into ornithine and polyamines in CAT2-deficient alternatively activated macrophages, suggesting the requirement for CAT2 for arginase activity in intact cells. In order to directly test the role of CAT2 in arginase activity, we used the whole cell arginase activity assay [15]. Importantly, as opposed to the assay used by Thompson et al. [9] and by us in lysed macrophages (Figure 5B) that assesses the arginase activity in ruptured cells or tissues and is thus primarily a reflection of the amount of arginase expression, the whole cell assay accounts for the role of trans-membrane transport for arginase function. We used peritoneal macrophages since we were unable to collect sufficient numbers of lung macrophages for this assay. Our data demonstrate a partial requirement for CAT2 for arginase activity in intact macrophages, suggesting either an alternative transporter or intracellular pool of arginine can contribute to arginase activity. This is in contrast to NOS activity, which depends on arginine transport from the extracellular space [15,28,29].

## Conclusions

In summary, we have described a pathway (involving CAT2 and arginine homeostasis) not previously examined in the context of lung inflammation and fibrosis. We find that while CAT2 is highly upregulated in experimental asthma, it does not have a role in allergic airway inflammation. Rather, CAT2 is critically important for interstitial lung fibrosis.

## Abbreviations

BALF: bronchoalveolar lavage fluid; CAT: cationic amino acid transporter; NOS: nitric oxide synthase; OVA: ovalbumin; STAT6: signal-transducer-and-activator-of-transcription 6

## Competing interests

The authors declare that they have no competing interests.

## Authors' contributions

KAN, MGC and NZ performed the research; LGE provided critical reagents (CAT2-deficient mice); MER participated in the conception and design of the study and helped revise the manuscript; LGE and MGC helped revise the manuscript; NZ participated in the conception, design and coordination of the study, analyzed the data and drafted the manuscript. All authors read and approved the final manuscript.
